# Removal of Cancer by the Knife

**Published:** 1844-07-27

**Authors:** 


### REMOVAL OF CANCER BY THE KNIFE.

M. Leroy d'Etiolles asserts, in a paper recently read before the Academy of Sciences, that extirpation, as far as it is practicable by surgical operation, does not stop the progress of this disease; and that, as a general rule, the knife should never be resorted to except for cancer affecting the lips and skin. In other cases, he says, extirpation should never be attempted, except when the life of the patient is placed in danger by hæmorrhage from ulceration. The same opinion is intertained upon this subject by the profession in Italy: at the recent meeting of the Scientific Congress at Lucca, the awarding of a a prize of £20, by the medical section, for the best Memoir on Cancer, gave rise to a lengthened debate on this formidable disease; in the course of which Professors Pacini, Cento, Canti, and Regnoli, maintained that it is an incurable affection. Dr. Regnoli read a statistical report which he had drawn up, and from which it appeared, that out of two hundred and fifty persons on whom cancerous degenerations had been extirpated by the knife, scarcely twenty had survived the third year. He was, therefore, induced to look upon amputation as a palliative measure only.—*Ibid.*

